# Supplementary figures and images for: Cryptic Diversity in Colombian Edible Leaf-Cutting Ants (Hymenoptera: Formicidae)
Source: Insects. 2018 Dec 12;9(4):191. doi: 10.3390/insects9040191 (PMC6316631; doi:10.3390/insects9040191)

| Maximum subproblem | SP-Score | PASTA-score |
|--------------------|----------|-------------|
| 5                  | 0.84     | -11610      |
| 10                 | 0.98     | -9357       |
| 15                 | 0.95     | -9885       |
| 20                 | 0.66     | -10058      |
| 25                 | 0.96     | -9128       |
| 30                 | 0.66     | -9627       |
| 35                 | 0.69     | -8939       |
| 40                 | 0.61     | -8994       |
| 45                 | 0.69     | -9032       |
| 50                 | 0.61     | -8998       |
| 55                 | 1.00     | -7848       |
| 60                 | 0.99     | -7942       |
| 65                 | 0.99     | -7948       |
| 70                 | 0.99     | -8135       |
| 75                 | 0.99     | -8147       |
| 80                 | 0.99     | -8711       |
| 85                 | 0.92     | -8801       |
| 90                 | 1.00     | -7996       |
| 95                 | 0.99     | -8292       |
| 100                | 1.00     | -7992       |
| 125                | 0.99     | -7936       |
| 150                | 0.99     | -7906       |
| 175                | 0.99     | -7914       |

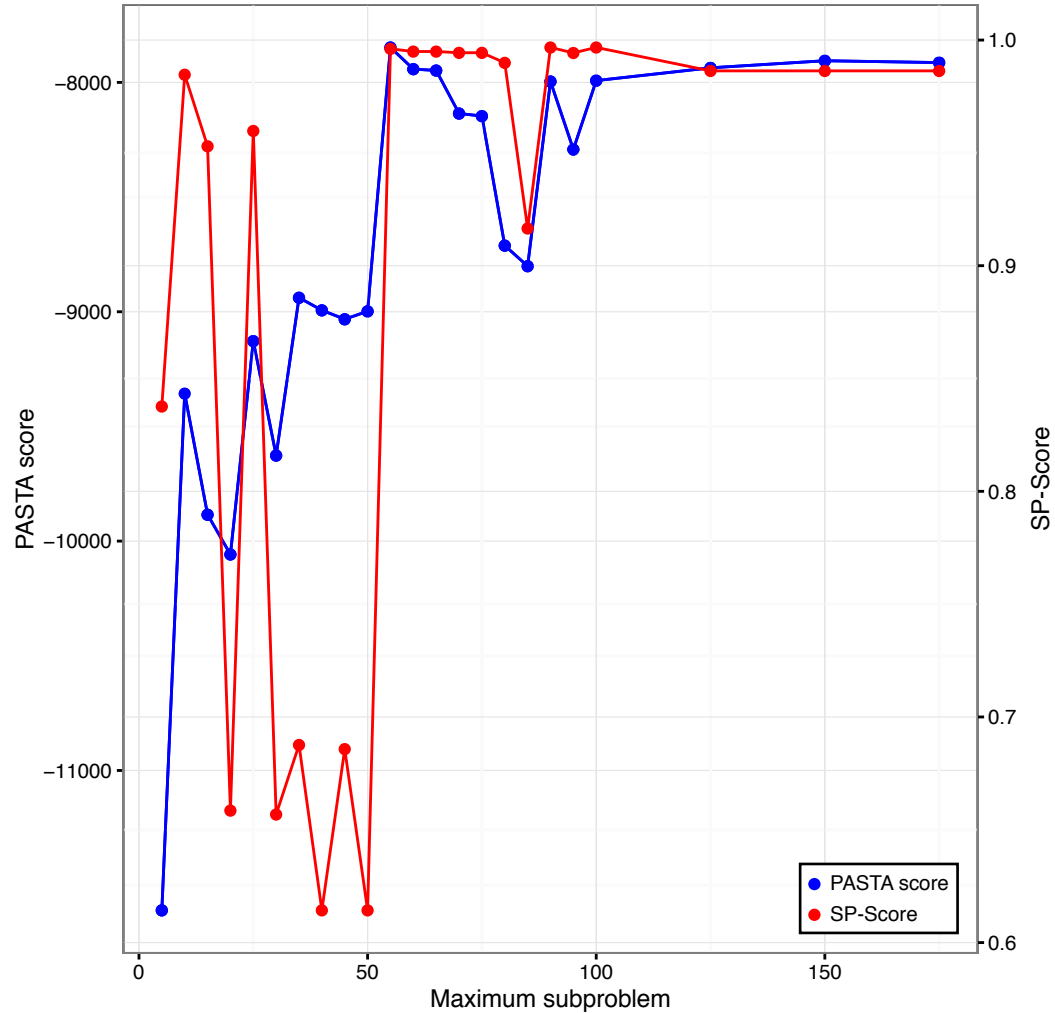

Supplement: Supplementary file 1 [file insects-09-00191-s001.zip › insects-388917-SI/insects-388917-s/Figure_S1.pdf]

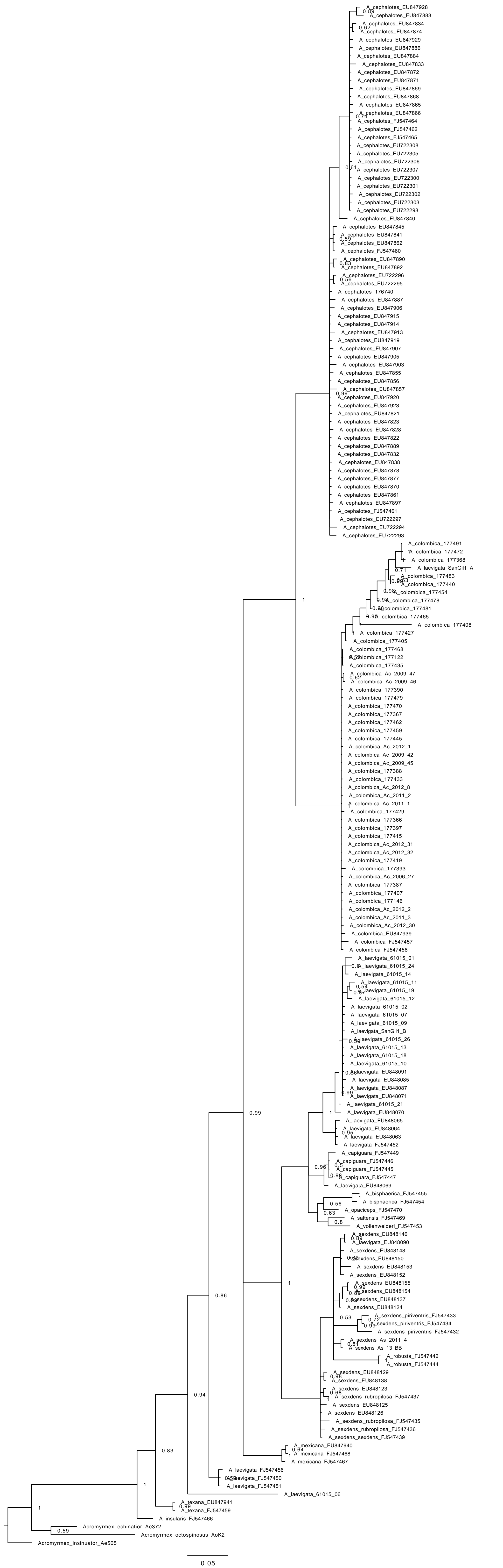

Supplement: Supplementary file 1 [file insects-09-00191-s001.zip › insects-388917-SI/insects-388917-s/Figure_S2.pdf]

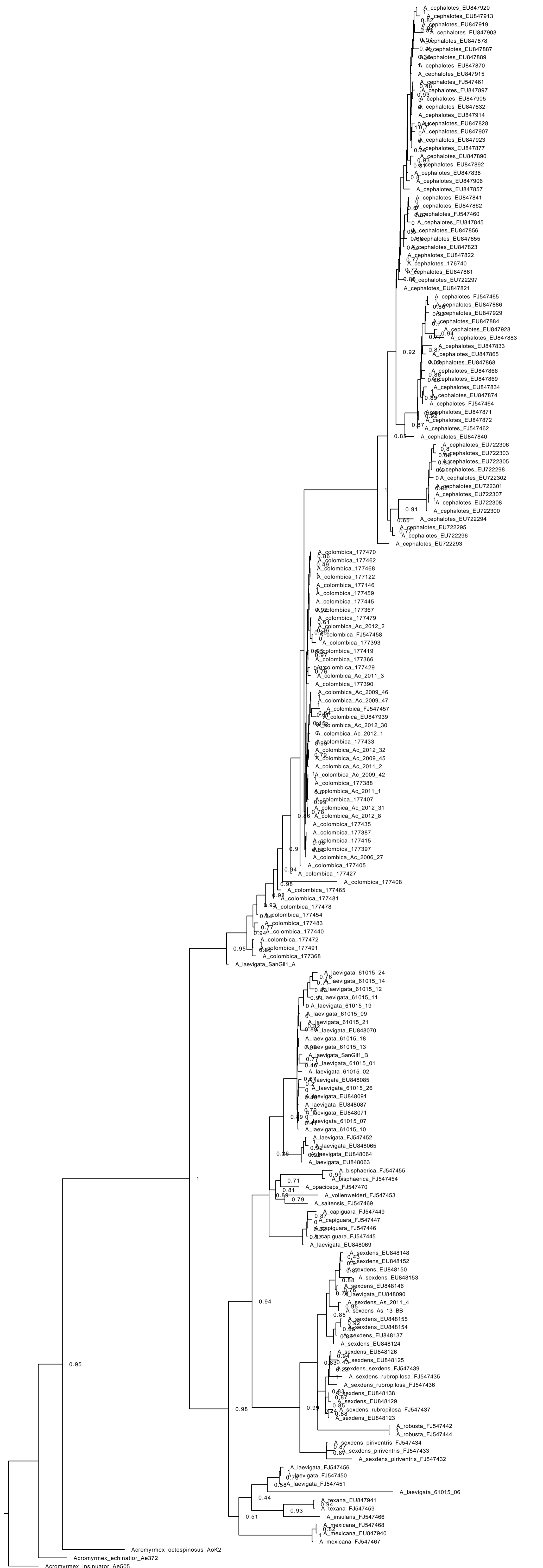

0.05

Supplement: Supplementary file 1 [file insects-09-00191-s001.zip › insects-388917-SI/insects-388917-s/Figure_S3.pdf]

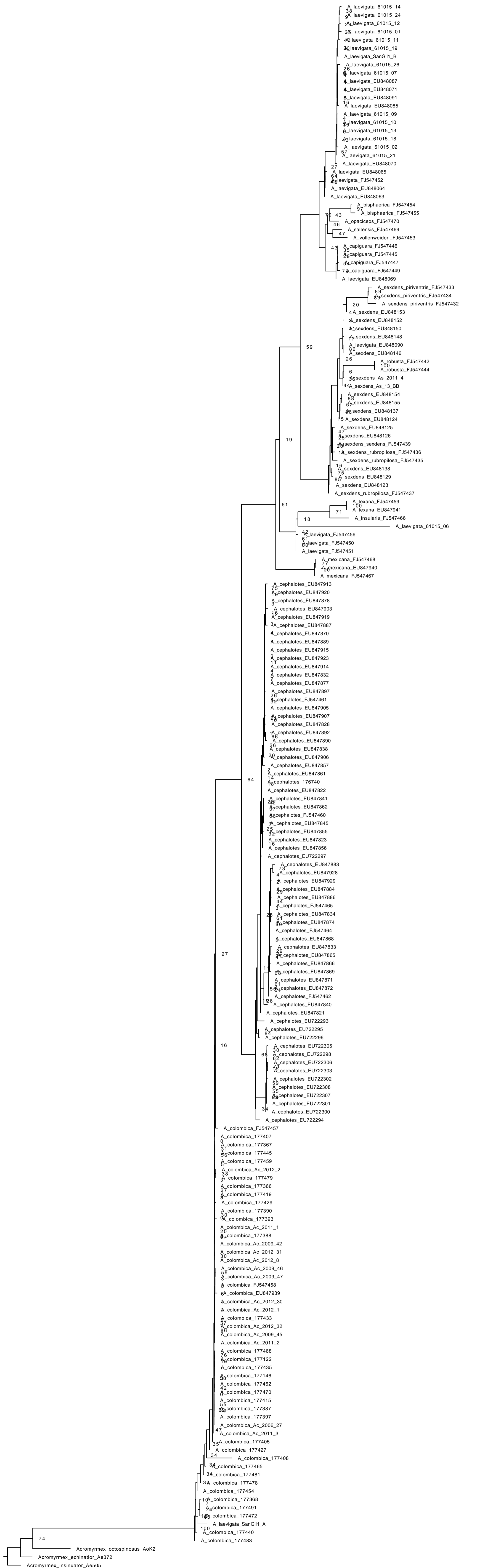

Supplement: Supplementary file 1 [file insects-09-00191-s001.zip › insects-388917-SI/insects-388917-s/Figure_S4.pdf]

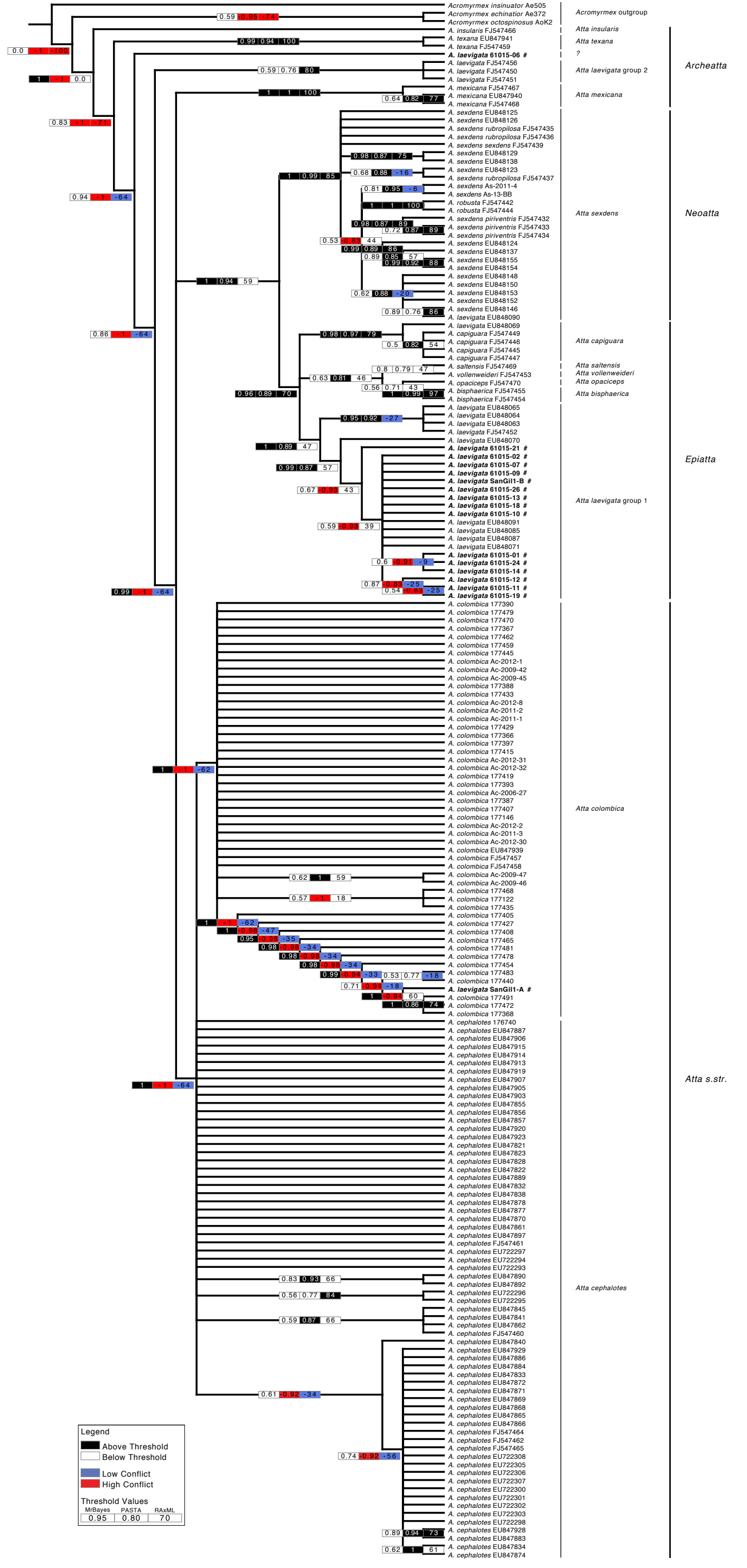

Supplement: Supplementary file 1 [file insects-09-00191-s001.zip › insects-388917-SI/insects-388917-s/Figure_S5_revised.pdf]
